# Temporal road closures improve habitat quality for wildlife

**DOI:** 10.1038/s41598-019-40581-y

**Published:** 2019-03-07

**Authors:** Jesse Whittington, Petah Low, Bill Hunt

**Affiliations:** Parks Canada Agency, Banff National Park Resource Conservation, Banff, Alberta Canada

## Abstract

Increasing levels of human activity threaten wildlife populations through direct mortality, habitat degradation, and habitat fragmentation. Area closures can improve habitat quality for wildlife, but may be difficult to achieve where tourism or other economic drivers are a priority. Temporal closures that limit human use during specific times of day have potential to increase habitat quality for wildlife, while continuing to provide opportunities for human use. However, the effectiveness of daily temporal closures has not been tested. We assessed how implementation of a temporal road closure affected wildlife movements in Banff National Park. Parks Canada closed a popular 17 km stretch of road between 2000 and 0800 hours to improve habitat quality for wildlife. We assessed the effectiveness of the closure on nine mammal species using three sets of data: remote cameras, road surveys, and grizzly bear (*Ursus arctos*) GPS data. In all three analyses, wildlife detection rates on the road doubled during the closure while remaining unchanged in reference areas. Our strong and consistent results suggest temporal closures are an important conservation tool that can increase habitat quality for wildlife while minimizing effects on people.

## Introduction

Increasing levels of human use threaten wildlife populations globally through increased mortality, habitat loss, and habitat fragmentation^1–3^. Road networks affect wildlife populations directly when animals are struck by vehicles^4^ and indirectly when they increase hunter access^5,6^ or obstruct movement and fragment wildlife populations^7,8^. Wildlife may avoid travelling near roads, trails, and towns to reduce their probability of encountering people^9–11^ or they may become more nocturnal near areas with people^12^. For example, numerous studies have found that grizzly bears (*Ursus arctos*) avoid high use roads^13,14^, become more nocturnal to avoid encounters with people^13,15^, incur elevated stress levels near human activity^16^, and have lower survival rates and densities in areas of high road density^5,17^. Conversely, grizzly bears and other species may select linear features with few people as easy travel routes^13,18,19^. Thus, roads and human activity can influence predator-prey dynamics by decreasing predation risk when wildlife prey species seek refuge from predators near people^20,21^ or increasing predation risk when linear features facilitate predator access and search efficiency^22,23^. However, increases in human activity have reduced wildlife movement worldwide^24^ and conservation actions are frequently required to reduce mortality, restore habitat quality, and improve connectivity.

Conservation actions that reduce anthropogenic effects on wildlife include establishment of protected areas^25,26^, de-activation of roads^5^, modifying resource extraction practices^27^, and establishment of wildlife corridors^28,29^. Even small-scale conservation actions have potential to improve habitat quality and connectivity in areas that are pinch points to movement or are located in critical habitat such as around den sites. For example, creation of wildlife crossing structures over highways in conjunction with highway fencing can reduce wildlife mortality by more than 80%^30^ and improve connectivity for wildlife^31^. Area closures are frequently used to reduce human activity and to improve habitat security^5,32^. Closures can be implemented permanently or can be strategically applied during specific seasons when they have the greatest conservation value. For example, Banff National Park frequently implements spring closures around wolf (*Canis lupus*) den sites near high visitation areas because wolf pups less than six weeks old are more susceptible to disturbance and den abandonment^33^. The timing of seasonal closures can be optimized based on the movement ecology and life-history of animals^34^. While effective, implementation of conservation actions like permanent or seasonal closures can be challenging when they negatively affect local businesses and opportunities for tourism, recreation, and resource extraction.

Temporal closures, which we define as closure of areas to human activity for a portion of the day, are a management tool that has potential to improve habitat quality for wildlife while minimizing effects on human activity and potentially alleviate loss of social capital^35^. Temporal closures may not be as effective as full closures, but in some cases may be the only option available depending on competing socio-economic management priorities. Many species adapt to human development by becoming more nocturnal and by travelling near developed areas at night to minimize encounters with people^36–39^. Similarly, detection rates and diurnal activity of leopards (*Panthera pardus*) increased in Thailand during a six month closure caused by flooding^40^. Thus, wildlife could potentially adapt to changes in human activity associated with temporal closures and increase their use of habitat near linear features. However, no studies to our knowledge have investigated wildlife responses to temporal closures.

We evaluated wildlife responses to a spring, night-time closure of a secondary road in Banff National Park of Canada. The number of visitors to Banff National Park has increased to over 4 million people per year (https://www.pc.gc.ca/en/docs/pc/attend) and high levels of human use, commercial development and a busy transportation network have reduced habitat quality and connectivity for wildlife^9,36^. Parks Canada implemented a temporal closure on a 17 km section of road to improve habitat quality and security for wildlife. The secondary road was closed for 12 hours a day during spring to provide wildlife with a predictable reprieve from human disturbance when many species emerge from their dens, give birth, and raise their offspring. Our objective was to determine whether wildlife use of the secondary road would increase during the temporal closure. We addressed this question using a combination of remote cameras, road-side observational surveys, and movement data from global position system (GPS) collared grizzly bears. We expected that wildlife use of the road would increase during the closure and that this would be reflected in increased detection of wildlife on remote cameras and road surveys, and a higher probability of road use by GPS collared bears.

## Methods

### Study Area

**Figure 1:**
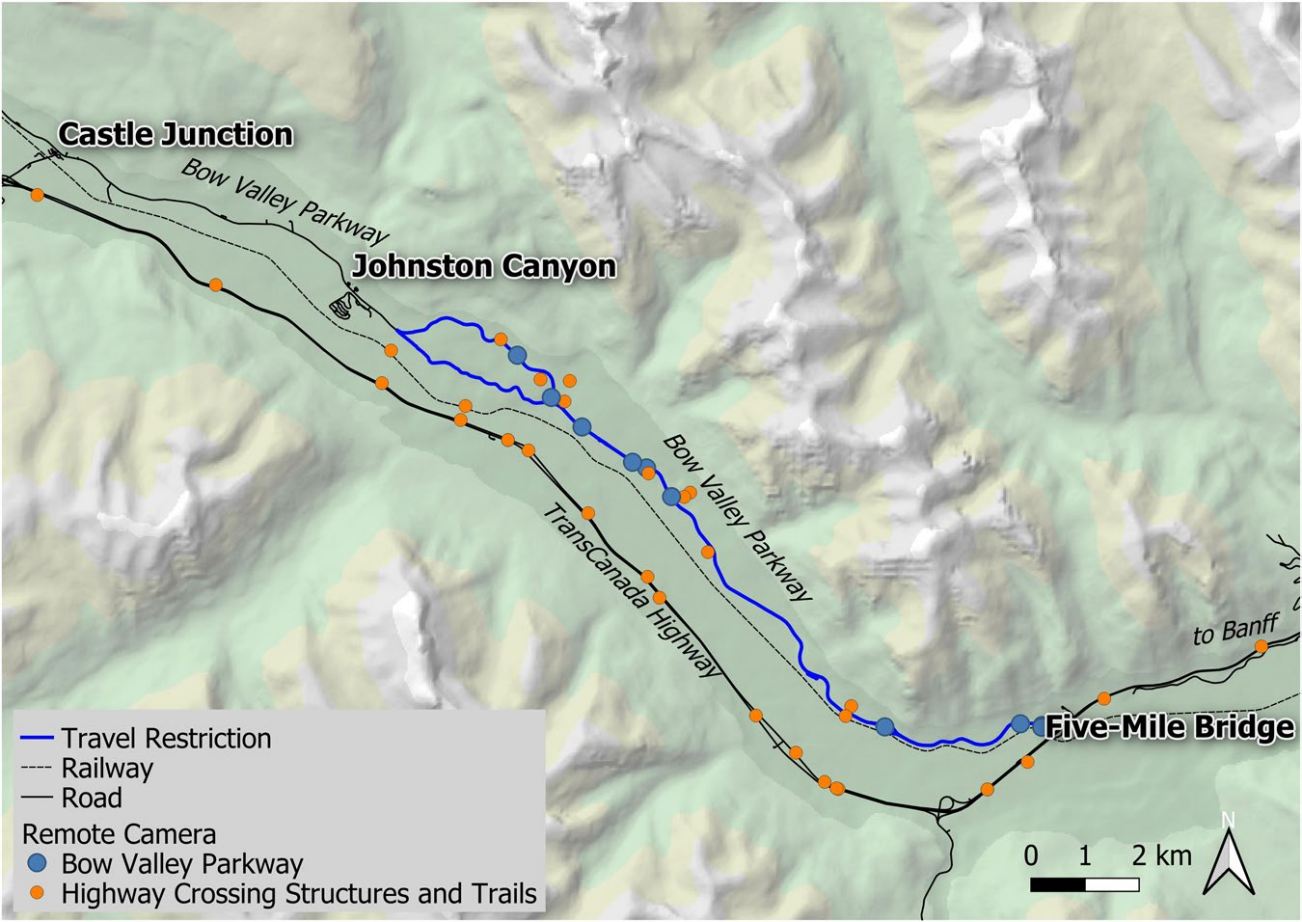
The night time travel restriction closed 17 km of the Bow Valley Parkway in Banff National Park between Johnston Canyon and Five-Mile Bridge from March 1 through June 25 between 2000 and 0800 hours. Remote cameras compared wildlife detections on ten road-based cameras to detections on 64 cameras distributed throughout the Bow Valley (reference data was collected at the same time but at different locations). Roadside observation surveys compared wildlife detections along the road within the temporal closure to detections on the permanently open road west of the closure to Castle Junction (reference data was collected at the same time but at a different location). Grizzly bear analysis selected GPS locations within 3.0 km of the temporal closure area and compared use of the Bow Valley Parkway prior to closure implementation (2012–2013) to use during the temporal closure (2014–2017; reference data was collected during different years with some different animals, but occurred within the same spatial area).

Our study occurred in the Bow Valley within Banff National Park, Alberta, Canada (Fig. 1). Banff National Park is characterised by rugged topography, cold winters, and cool summers^41^. The temporal closure occurred within the montane ecoregion, which contains the highest quality habitat for many species because it occurs along low elevation valley bottoms and has warmer temperatures, earlier springs, and higher plant productivity compared to other ecoregions. The montane ecoregion contains small meadows nested within lodgepole pine (*Pinus contorta*), Douglas-fir (*Pseudotsuga menziesii*), white spruce (*Picea glauca*), Engelmann spruce (*P*. *engelmannii*), and aspen (*Populus tremuloides*) forests. The Bow Valley Parkway transitions into the lower subalpine ecoregion west of the temporal closure, where the spatial coverage lodgepole pine and Engelmann spruce increases while the spatial coverage of aspen and open meadows decreases.

The Bow Valley Parkway is a 48 km secondary road between the town of Banff and the hamlet of Lake Louise, Alberta. The Bow Valley Parkway parallels a national railway, the Bow River, and the TransCanada Highway which occurs on the opposite side of the valley (Fig. 1). Vehicle traffic on the Bow Valley Parkway almost doubled from 297,840 vehicles in 2007 to 437,061 vehicles in 2017 with most traffic occurring from May through September (Parks Canada unpublished data). Parks Canada initiated a mandatory seasonal closure along the eastern section of the Bow Valley Parkway in 2014 to improve habitat security for wildlife. The temporal closure applied to a 17 km section of the Parkway, from March 1 to June 25 between 2000 and 0800 hours (night time), during which time the area was closed to all motor vehicle, bicycle and foot traffic. This 17 km section of the Bow Valley Parkway was selected for the temporal closure because it contained the highest quality spring habitat for many large mammals, wolves denned in the area, and the area did not contain campsites or commercial accommodation. Prior to implementation of the closure, 6% of the vehicle traffic occurred at night between 2000 and 0800 hours (15,647 of 255,565 vehicles recorded from March through June, 2010–2012). An average of 47 vehicles per day (range = 1 to 1026) travelled during the night time hours and 716 vehicles per day (range = 61 to 3851) during daylight hours.

### Study Design

We assessed the effects of the temporal closure on wildlife movements using three methods: remote cameras, roadside observational surveys, and GPS collared grizzly bears. For each method we compared wildlife detections along the restricted section of the Bow Valley Parkway (Treatment = 1) to reference data (Treatment = 0). Reference data was collected during the same time but at different locations for remote cameras and roadside surveys, and on different years but within the same spatial area for grizzly bear GPS data. We compared wildlife detection rates when the temporal closure was in effect (Closed Time = 1) to when the road was open (Closed Time = 0) at both treatment and reference sites. Thus, we assessed the effectiveness of the closure using a before-after control-impact type study design, albeit with a sample size of one closure. We provide all data used for the analysis in Data Dryad Digital Repository (10.5061/dryad.5cv05bc).

### Remote Cameras

We set up ten remote cameras along the restricted section of the Bow Valley Parkway road (Treatment Area = 1, Fig. 1). We compared wildlife detection rates at those cameras to 54 reference cameras (Treatment Area = 0) including 18 cameras on trails and 36 on highway crossing structures. These cameras were a subset of Parks Canada’s broader remote camera network^42,43^. We selected remote cameras in the Bow Valley that were within 100 m elevation of the Bow Valley Parkway (1402 m in central region) to minimize confounding effects of elevation on detection probability. The highway crossing structures were located on the TransCanada Highway, were closed to the public, and were frequently used by carnivores and ungulates^43,44^. We selected data from 2014 through 2017 between March 1 and June 25, which is the period when the night time vehicle closure was implemented. We used covert motion triggered cameras (Reconyx Hyperfire PC900 Professional cameras, Holmen, Wisconsin).

We compared hourly wildlife detection rates when the road was closed (0700–0800 and 2000–2100, Closed Time = 1) to when the road was open (0800–0900 and 1900–2000, Closed Time = 0). We selected data within one hour of the road opening and closing because road based remote cameras were programmed to turn off from 0900 to 1900 to prevent challenges associated with classifying thousands of vehicle images. We determined for each camera sampling day and hour whether or not at least one wildlife species was detected. Wildlife species included red fox (*Vulpes vulpes*), cougar (*Puma concolor*), coyote (*Canis latrans*), wolf, black bear (*Ursus americanus*), grizzly bear, bighorn sheep (*Ovis canadensis*), mule deer (*Odocoileus hemionus*), white-tailed deer (*Odocoileus virginianus*), elk (*Cervus canadensis*), and moose (*Alces alces*). We analysed the data using a generalized linear mixed effects model with a binomial link. Explanatory variables included Treatment Area, Closed Time, and the interaction between Treatment Area and Closed Time. The main parameter of interest was how detection probability in the two areas changed when the road was closed. Changes in the reference area could be attributed to natural changes in diurnal activity. Additional changes in the treatment area, which was estimated with the interaction between Treatment Area and Closed Time, could be attributed to increases in human activity. We included additional parameters to address spatial variation in wildlife detection rates. Habitat related covariates such as shrub, grassland, deciduous, and coniferous forests were poor predictors of detection probability in preliminary analyses. However, we included days since May 1 as linear and quadratic terms to account for seasonal trends in wildlife detection rates; hours since sunrise and to sunset (SunRiseSet) to account for diurnal patterns of wildlife activity with positive values reflecting daylight and negative values reflecting darkness; highway overpasses and open span underpasses where wildlife have higher probability of detection compared to smaller underpasses and trails^43^; and elevation (m) which affects wildlife distribution in the spring. More specifically, we calculated SunRiseSet as morning * (*t* − *t*_*sunrise*_) + (1 − *morning*) * (*t*_*sunset*_ − *t*) where *morning* equalled 1 for morning surveys and 0 for evening surveys, *t* was the time of day, *t*_*sunrise*_ was the time of sunrise, and *t*_*sunset*_ was the time of sunset. We included random effects for camera location to account for repeated measures at individual camera locations. We centred linear and quadratic measures of days since May 1, SunRiseSet, and elevation based on their mean and standard deviation to improve convergence and interpretability^45^. We selected the most parsimonious model here and below by comparing all covariate combinations using Akaike Information Criterion (AIC_c_) corrected for small sample size^46^ using the program R version 3.5.1^47^ and the packages glmmTMB^48^ and MuMIn^49^. We reported all models within 2 AIC_c_ of the top-ranked model and provided parameter estimates from the top ranked model.

### Road Surveys

Technicians conducted driving surveys for wildlife along the restricted 17 km section of the Bow Valley Parkway (Treatment Area = 1) and along an adjacent permanently open 7 km section of the Bow Valley Parkway (Treatment Area = 0). The treatment area occurred fully within the montane ecoregion while the reference area transitioned from the montane to the lower subalpine ecoregion. The treatment area contained more prescribed burns than the reference area and contained higher quality habitat for many species. Surveyors started road side wildlife surveys one hour prior to the Bow Valley Parkway opening in the morning or closing at night. Surveyors then repeated the survey once the road changed from closed to open in the mornings and from open to closed in the evenings. The return survey started an average of 35 minutes after the road changed from closed to open or from open to closed. If wildlife disturbance by surveyor presence on the first survey reduced wildlife detection rates on the return survey, then surveyor disturbance could positively bias effects of the morning surveys and negatively bias effects of the evening surveys. Surveyors minimized stopping time and remained in their vehicles to reduce this confounding effect. Sixty-seven surveys were conducted from February 18 through July 1 during 2014–2016; 59 surveys were conducted during the restricted activity period (March 1 through June 25) and 8 surveys before or after the restricted activity period when the entire road was open day and night. Most (n = 63) of the surveys occurred in the morning, because night-time surveys were potentially confounded by vehicles that lingered in the closure beyond 2000 hrs. The remote camera data suggested that more vehicle traffic occurred one hour after the road was closed at night (mean = 0.40 vehicles per day, range = 0 to 14) compared to one hour prior to road opening in the morning (mean = 0.02 vehicles per day, range = 0 to 3). These traffic volumes were much lower than daytime traffic volumes, but they potentially reduced the effectiveness of the temporal closure.

We tallied the number of wildlife detections for each survey, section of road, and time period. We simplified the number of detections to wildlife presence-absence because 86% of the records had zero or one detections. We analysed the data using generalized linear models with a binomial link and a random effect for survey (date and morning/evening). Explanatory variables included the effects of Treatment Area, Closed Time (0 = 0800 to 2000; 1 = 2000 to 0800), the interaction between Treatment Area and Closed Time, hours since sunrise and to sunset (SunRiseSet) because some animals may have been more active during dawn and dusk, and days since May 1 as a linear and quadratic term to accommodate seasonal changes in wildlife use. We centred SunRiseSet and days since May 1 based on their mean and standard deviation. We compared all covariate combinations using AIC_*c*_. We selected all models within 2 AIC_c_ of the top-ranked model and reported parameter estimates for the top-ranked model.

### Grizzly GPS

We used GPS collared grizzly bear data to determine how the temporal closure affected grizzly bear selection for the Bow Valley Parkway. From 2012–2016, Parks Canada in collaboration with Canadian Pacific Railway and the University of Alberta collected grizzly bear GPS data to understand root causes of grizzly bear mortality on the railway (Parks Canada Research Collection Permit LL – 2012-010975). We selected GPS data from grizzly bears that used the restricted section of the Bow Valley from 2012 to 2017. We limited the data from March 1 through June 25. We then filtered GPS data to 2 hour fix intervals. We selected grizzly bear steps when the Bow Valley Parkway was available to the grizzly bears, defined as instances where both the start and end locations were within 3.0 km of the Bow Valley Parkway because 3.0 km was the 95th percentile of 2 hour movements. We used the spatial attributes of the end location for analysis.

We defined our response variable as whether or not each GPS location was within 30 m of the Bow Valley Parkway. We included covariates for days since May 1, hour of day (sine and cosine transformed), Treatment Year (0 = 2012–2013, 1 = 2014–2017), Closed Time (0 = 0800 to 2000; 1 = 2000 to 0800), the interaction between Treatment Year and Closed Time, and a random effect for individual animal. We scaled days since May 1 scaled by its mean and standard deviation. We compared all model combinations and selected the top ranked model as the model with the lowest AIC_c_.

## Results

### Remote Cameras

**Figure 2:**
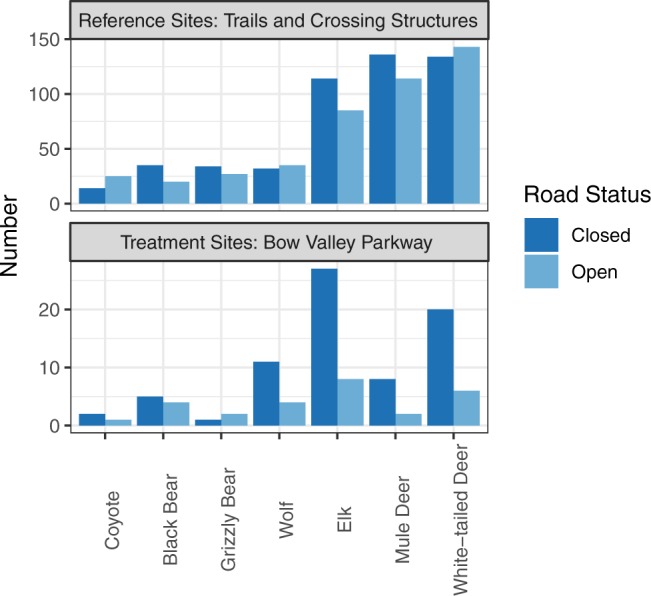
Number of wildlife detections from remote cameras within one hour of the temporal closure on Bow Valley Parkway opening in the morning and closing at night. Cameras were located along the Bow Valley Parkway road (n = 10) and at reference sites on trails and highway crossing structures (n = 54).

Remote cameras compared wildlife detections on ten road-based cameras to detections on 64 cameras distributed throughout the Bow Valley. Reference data was collected at same time but at different spatial locations. The roadside and trail cameras operated for 2,756 and 13,312 camera sampling days, respectively, from 2014 to 2017 between March 1 and June 25. The roadside cameras operated from 1900 to 0900 hours. Roadside and trail cameras recorded 171 and 1686 detections of wildlife respectively between the hours of 1900–2100 and 0700–0900 (Fig. 2). Deer and elk were the most prevalent ungulate species, whereas wolves were the most commonly detected carnivore. Species varied in their responses to the road opening. Wolves, deer, and elk use of the Bow Valley Parkway decreased the most when the road changed from closed to open. Grizzly bear detections increased on the road but declined on the reference sites, however sample sizes were likely too small to be statistically significant in single species models as fewer than 5 detections occurred on the road (Fig. 2). The number of detections for each species was likely influenced by individual tolerance for human activity coupled with population density, home range size, movement rates, and habitat selection.

**Figure 3:**
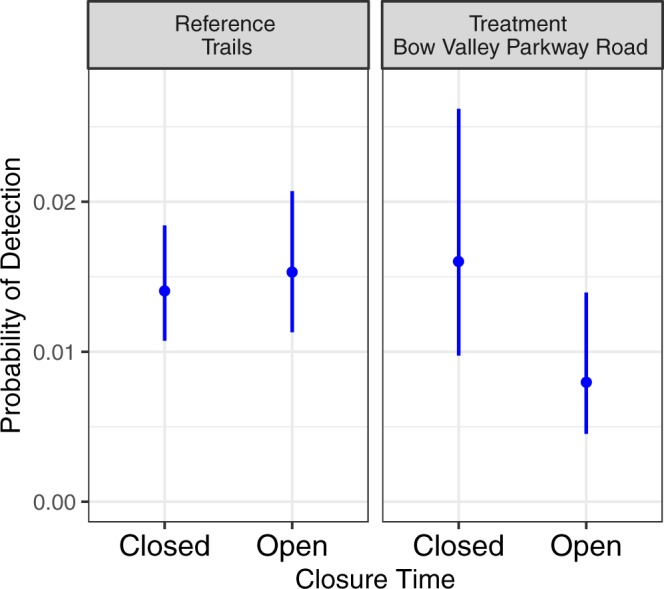
Hourly probability of detecting wildlife and 95% CI’s on remote cameras as a function of location and status of the temporal closure.

The top mixed effects logistic regression model contained covariates for Treatment Area, Closed Time, the interaction between Treatment Area and Closed Time, elevation, TransCanada highway overpass and open span underpass, SunRiseSet, and linear and quadratic terms for days since May 1 (Table 1). Only one other model was within 2 ΔAIC_c_ of the top ranked model. The second model included the same covariates as the top ranked model but lacked SunRiseSet. Wildlife detections rates doubled during the temporal closure on the restricted section of the Bow Valley Parkway but remained unchanged at reference locations (Table 2, Fig. 3). Detection rates increased at lower elevations and were higher on highway overpasses and open span underpasses compared to cameras on trails and small highway underpasses. Detection rates on trails were similar to detection rates on the parkway when it was closed. Wildlife detection rates increased throughout the spring then plateaued in June. Wildlife detection rates marginally decreased with time since SunRiseSet.

**Table 1:** Model selection tables for wildlife detections on remote cameras, on roadside surveys, and for grizzly bear selection of the Bow Valley Parkway. Generalized linear mixed effects models were used for all analyses.

Model	Parameters	df	AIC_c_	ΔAIC_c_	Akaike Weight
Remote Camera	ClosedTime + Elev + May1 + May1^2^ + HwyOver + TreatmentArea + ClosedTime:TreatmentArea + SunRiseSet	11	13779.9	0.0	0.55
	ClosedTime + Elev + May1 + May1^2 ^+ HwyOver + TreatmentArea + ClosedTime:TreatmentArea	10	13780.3	0.4	0.45
	Null Model	3	14390.7	610.9	0.00
Road Survey	ClosedTime + May1 + May1^2^ + TreatmentArea + ClosedTime:TreatmentArea	7	214.2	0.0	0.33
	ClosedTime + May1 + May1^2^ + TreatmentArea + SunRiseSet	7	214.6	0.4	0.27
	ClosedTime + May1 + May1^2^ + TreatmentArea + SunRiseSet + ClosedTime:TreatmentArea	8	215.2	1.0	0.20
	ClosedTime + May1 + May1^2^ + TreatmentArea	6	215.2	1.0	0.20
	Null Model	2	258.6	44.4	0.00
Grizzly GPS	May1 + ClosedTime + TreatmentYear + ClosedTime:TreatmentYear	6	575.5	0.0	0.38
	May1	3	576.3	0.8	0.26
	May1 + ClosedTime	4	576.7	1.2	0.21
	May1 + ClosedTime + TreatmentYear + ClosedTime:TreatmentYear + HourSin	7	577.3	1.9	0.15
	Null Model	2	609.9	34.4	0.00

The table shows the null model and all models within 2 AIC_c_ of the top model.

**Table 2:** Parameter estimates for wildlife detections on remote cameras, on roadside surveys, and for grizzly bear selection of the Bow Valley Parkway. Generalized linear mixed effects models were used for all analyses.

Model	Parameter	Estimate	SE	p value
Remote Camera	Intercept	−4.164	0.157	<0.001
	ClosedTime	−0.086	0.132	0.518
	Elevation	−0.313	0.095	0.001
	HwyOverpassOpenspan	1.002	0.211	<0.001
	DaysMay1	0.894	0.114	<0.001
	DaysMay1^2^	−0.268	0.042	<0.001
	SunRiseSet	−0.198	0.128	0.121
	TreatmentArea	−0.662	0.305	0.030
	TreatmentArea:ClosedTime	0.793	0.187	<0.001
Road Survey	Intercept	−1.671	0.662	0.012
	ClosedTime	0.333	0.756	0.659
	DaysMay1	0.590	0.224	0.008
	DaysMay1^2^	−0.439	0.178	0.014
	TreatmentArea	0.659	0.693	0.342
	TreatmentArea:ClosedTime	1.614	0.879	0.066
Grizzly GPS	Intercept	−3.667	0.440	<0.001
	ClosedTime	−0.226	0.355	0.525
	DaysMay1	1.103	0.212	<0.001
	TreatmentYear	−0.303	0.568	0.593
	TreatmentYear:ClosedTime	1.107	0.517	0.032

### Road Surveys

Roadside observation surveys compared wildlife detections along the road within the temporal closure to detections on the permanently open road west of the closure. Reference data was collected at same time but at a different spatial location. We recorded 140 detections of wildlife on the 67 surveys including four grizzly bears, six black bears, fifteen wolves, 49 deer, and 58 elk. We recorded 112 wildlife detections on the restricted section of the road when it was closed, 16 detections on the restricted section of the road when it was open, and 12 detections on the permanently open section of road.

**Figure 4:**
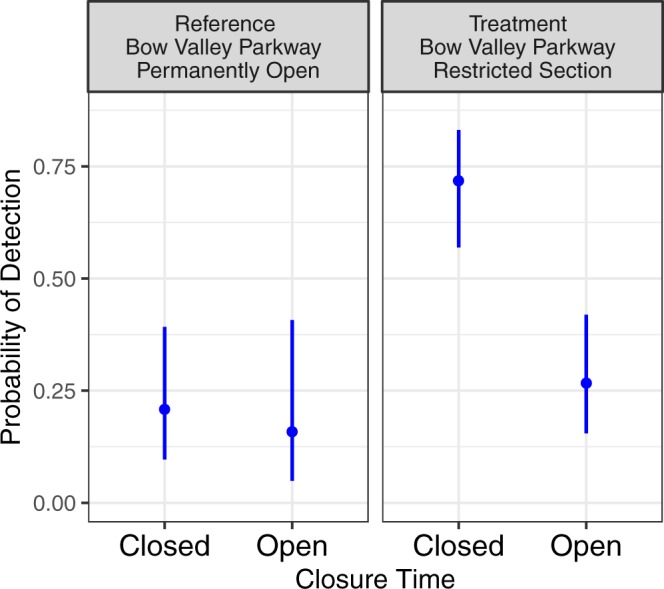
Probability of detecting wildlife and 95% CI’s on road-based observational surveys as a function of treatment area and status of the temporal closure.

The top logistic regression model for wildlife detections with a random effect for survey included linear and quadratic terms for days since May 1 and the interaction between Treatment Area and Closed Time (Table 1). Treatment Area, Closed Time, plus linear and quadratic terms for days since May 1 occurred in all four models within 2 ΔAIC_c_ of the top ranked model. Models with lower AIC_c_ scores either added terms for SunRiseSet or dropped the interaction between Treatment Area and Closed Time. Wildlife detection rates in the top ranked model more than doubled during the temporal closure in the treatment area but remained unchanged in the reference area (Table 2, Fig. 4). Detection rates increased during the spring and peaked during mid-May.

### Grizzly GPS

**Figure 5:**
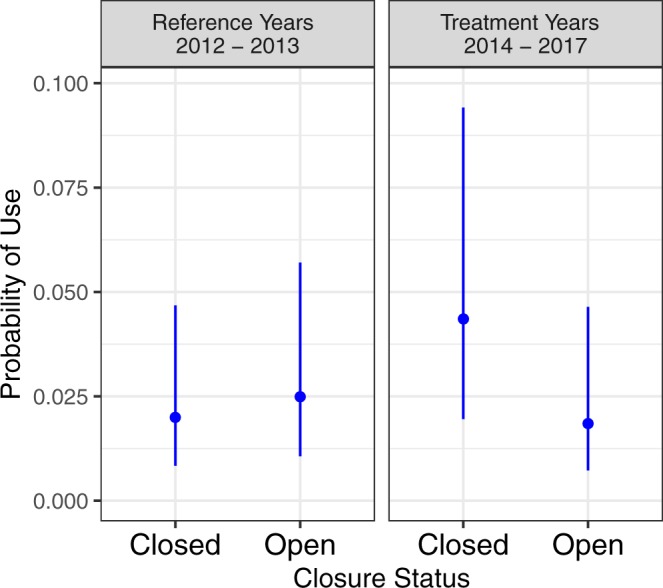
Grizzly bear probability of use and 95% CI’s on the Bow Valley Parkway as a function of treatment period and status of the temporal closure.

The grizzly bear analysis selected GPS locations within 3.0 km of the temporal closure area from March 1 through June 25 and compared use of the Bow Valley Parkway prior to closure implementation (2012–2013) to use during the temporal closure (2014–2017). Reference data was collected during different years and included different animals, but occurred in the same spatial area. Our analysis for grizzly bear selection of the Bow Valley Parkway used GPS data from 11 grizzly bears including seven bears from 2012–2013 and six bears from 2014–2017. Two of the grizzly bears had GPS data for both the reference and treatment years. Less than 3% of the GPS locations (71 of 2471 locations) occurred within 30 m of the restricted section of the Bow Valley Parkway. The top candidate model contained covariates for days since May 1, Treatment Year, Closed Time, and the interaction between Treatment Year and Closed Time (Table 1). All four models within 2 ΔAIC_c_ of the top model contained the terms DaysMay1. Lower ranked models added terms for hour of day (sine transformed) or dropped terms for Treatment Year, Closed Time, and/or the Treatment Year:Closed Time interaction. Grizzly bear selection for the road increased during the temporal closure during the treatment years (2014–2017) but remained unchanged during the reference years (2012–2013) (Table 2, Fig. 5). Grizzly bear selection for the road increased through the spring.

## Discussion

Our study clearly demonstrated, using multiple sources of evidence, that temporal closures can be used to restore habitat quality and improve connectivity. Three independent sets of data consistently found that ungulate and carnivore use of roads doubled when it was closed to vehicle traffic compared to when it was open. When the road was closed, wildlife detection rates on remote cameras increased on the road, while remaining unchanged at reference sites on wildlife trails and highway crossing structures. Similarly, wildlife detections during roadside observational surveys increased when the road was closed. GPS collared grizzly bears increased selection for the road when it was closed, while use remained unchanged during reference years when the road was permanently open. While temporal closures may not be as effective as full closures, our results provide evidence that they can alleviate the effects of human activity on wildlife. Prior to implementation of the temporal closure, most human activity occurred during the day. The temporal closure, which covered night and crepuscular hours, affected relatively few people but substantially improved habitat quality for wildlife. In this way, temporal closures offer managers a valuable tool for balancing the often conflicting mandates of species conservation and human use^25^.

Our study was the first to our knowledge that examined wildlife responses to temporal closures. However, our results were consistent with the growing body of research that shows many species avoid people by both avoiding high use areas^11,39^ and by modifying their temporal movement patterns so that they travel through developed areas when people are inactive^12,36,38–40^. Application of full or temporal closures can be most effective when they provide wildlife with increased habitat security during critical times of the year with limited forage or during denning and calving season^34^. Our study also further emphasizes that many wildlife avoid people on linear features rather than the physical characteristics of linear features. This is consistent with other research that found grizzly bears select roads with less than 20 vehicles per day, moderately avoided roads with 20–100 vehicles per day, and strongly avoided roads with >100 vehicles per day^13^. Similarly, black bears^19^ and wolves^50^ had higher detection rates on closed or low-use roads compared to open or high-use roads. That said, we recognize that even low-use linear features can affect search efficiency of predators^23^ and survival rates of prey^51^.

The main limitations of our research were that it lacked spatial replication, variation in the timing of temporal closures, and before-after data was correlated with time of day. Our study assessed the effects of a single road closure on the movements of wildlife. The level of inference would be strengthened by measuring wildlife responses to temporal closures at more sites. Similarly, the timing of the closure in our study was static between 2000 and 0800 hours to balance the needs of wildlife and people. Thus, our results could have been influenced by natural diurnal variation in wildlife movements, spatial variation in habitat quality, and individual variation in tolerance for human activity. Replicating closures to occur during different times of day and segments of road would strengthen confidence in our results. Moreover, such a study could assess how closure effectiveness changes with time of day. We addressed these limitations by using three independent data sets, using before-after control-impact analyses, and by including covariates like time of day and season in the analyses. The remote camera and road surveys occurred within one hour of the road closing or opening, which gave wildlife little time to adapt to the closure. Results might have been stronger had the surveys occurred midway through each period. Even so we found a positive effect of the temporal closure on wildlife detections. We grouped wildlife species together in our analyses because some species lacked adequate sample sizes. Ungulates and carnivores often respond differently to human-use^21^ and our combined analysis may have masked the responses of more wary species. Additional research into the effectiveness of temporal closures would help clarify how different species respond to temporal closures.

Temporal closures provide land-use managers with one of many conservation tools. Their effectiveness likely depends on the amount of high quality habitat, human use, and natural fragmentation in the surrounding landscape^52^. For example, female grizzly bear survival is influenced by both open road density and the amount of high quality secure habitat greater than 500 m from roads^53^. The positive effects of the temporal closure in our study likely occurred because our study occurred within a protected area along a road nested within high quality habitat and a broad assemblage of wildlife species. However, further research is required to better understand how the surrounding network of human activity and habitat quality affect the effectiveness of temporal closures. Temporal closures may not benefit wildlife in areas surrounded by high levels of human use and poor quality habitat^8,18^ or in areas where hunting threatens wildlife^5^. Similarly, temporal closures may not benefit animals with diurnal activity patterns that differ from the timing of the temporal closure^39^. Thus, full closures may be required to increase wildlife use in many situations.

## Conclusion

Increasing levels of human activity throughout the world can negatively affect wildlife movement^24^, distribution^54^, and biodiversity^2^. A variety of conservation tools ranging from the establishment of wildlife corridors to formation of protected areas are required to ameliorate the effects of human activity on wildlife. Temporal closures are an important conservation tool that can increase habitat quality and connectivity for wildlife while minimizing effects on people.

## Data Availability

Data and R scripts used for this paper are available at the Data Dryad Digital Repository 10.5061/dryad.5cv05bc.
